# Effect of Soluble Sulfide on the Activity of Luminescent Bacteria

**DOI:** 10.3390/molecules17056046

**Published:** 2012-05-21

**Authors:** Ying Shao, Ling-Ling Wu, Hong-Wen Gao, Feng Wang

**Affiliations:** 1 Key Laboratory of Yangtze River Water Environment, Ministry of Education, College of Environmental Science and Engineering, Tongji University, Shanghai 20092, China; E-Mails: 6598sy@tongji.edu.cn (Y.S.); hjwangfeng@tongji.edu.cn (F.W.); 2 State Key Laboratory of Environmental Pollution and Resource Reuse, College of Environmental Science and Engineering, Tongji University, Shanghai 200092, China; E-Mail: EMSL@tongji.edu.cn

**Keywords:** sulfide solution, light emission, inhibition ratios, EC_50_, hormesis-effect

## Abstract

Sulfide is an important water pollutant widely found in industrial waste water that has attracted much attention. S^2−^, as a weak acidic anion, is easy hydrolyzed to HS^−^ and H_2_S in aqueous solution. In this study, biological tests were performed to establish the toxicity of sulfide solutions on luminescent bacteria. Considering the sulfide solution was contained three substances—S^2−^, HS^−^ and H_2_S—the toxicity test was performed at different pH values to investigate which form of sulfide increased light emission and which reduced light emission. It was shown that the EC_50_ values were close at pH 7.4, 8.0 and 9.0 which were higher than pH 5 and 10. The light emission and sulfide concentrations displayed an inverse exponential dose-response relationship within a certain concentration range at pH 5, 6.5 and 10. The same phenomenon occurred for the high concentration of sulfide at pH 7.4, 8 and 9, in which the concentration of sulfide was HS^−^ >> H_2_S > S^2−^. An opposite hormesis-effect appeared at the low concentrations of sulfide.

## 1. Introduction

Sulfide, a serious environmental problem resulting from the oxidative dissolution of sulfide-rich mine tailings and coal burning, has been the focus of numerous studies and several comprehensive reviews [1,2]. It is flammable and explosive and its aqueous solution is alkaline, corrosive and an irritant. Sulfide, as a molecule, is toxic [3]. Sulfide-rich environments have been shown to interfere with metabolism, ion uptake, and growth of *Spartina alterniflora* Loisel and other salt marsh plants [4,5,6,7]. It inhibits mitochondrial cytochrome oxidase at submicromolar concentrations [3]. Other studies about insoluble sulfide show that sulfide clearly reduces ATP formation by its adverse effects on energy metabolism. Alcohol dehydrogenase activity is immediately reduced in the presence of sulfide. Moreover, the post-hypoxic cytochrome pathway is inhibited. The remaining electron flow may be directed towards an alternative pathway which does not generate any ATP after the branching point [8,9,10]. Overall metabolic activity is continually decreased because of the loss of adenylates with increasing time and sulfide concentrations. Under such conditions, tissues will gradually lose their viability [11].

However, there is limited or no specific information about the effects of the soluble sulfide on organisms. One of possible reasons maybe that sulfide is easily oxidized by oxygen in water and air [7], which conflicted with the living conditions of most organisms used in the toxicity tests. The other possible reason maybe that in sulfide solution three substances S^2−^, HS^−^ and H_2_S exist together, and it was difficult to identify which substance(s) induce the sulfide solution toxicity [12]. Some authors used different solutions of NaHS [13] or more often Na_2_S and implied that HS^−^ was the toxic component [14]. Other authors thought that the toxicity of sulfide solution mostly was due to hydrogen sulfide (H_2_S) because H_2_S is known as a very poisonous gas that, for example, leads to pulmonary edema when absorpted by breath [15], and that often was found in sewage treatment plants and under other anoxic conditions [16].

In this study, the luminous bacterium *Vibrio qinghaiensis* sp. Q67 was employed to evaluate the effect of sulfide solutions and to differentiate the different sulfides and their role. The luminescent bacterium Q67, a freshwater bacterium, can tolerate a wide range of pH values [17], and has been extensively used to assess the potential toxicity of different types of chemical pollutants [18,19,20,21]. Use of *V. qinghaiensis* sp. Q67 to evaluate the toxicity of secondary metabolites produced by microorganisms has therefore been suggested [17]. In addition, the toxicity test method based on luminous bacteria, which is also called Microtox, is very quick (the reaction time is only 15 min) [22], simple and sensitive for toxicity determination [23], and this could avoid the oxidization of sulfide to some extent. Even though the sulfide solutions contain three substances: S^2−^, HS^−^ and H_2_S, H_2_S dominates in acidic solutions and HS^−^ dominates in alkaline solutions. The toxicity test was performed at different pH values to investigate which form of sulphur induced the sulfide solution toxicity.

## 2. Results and Discussion

**Figure 1:**
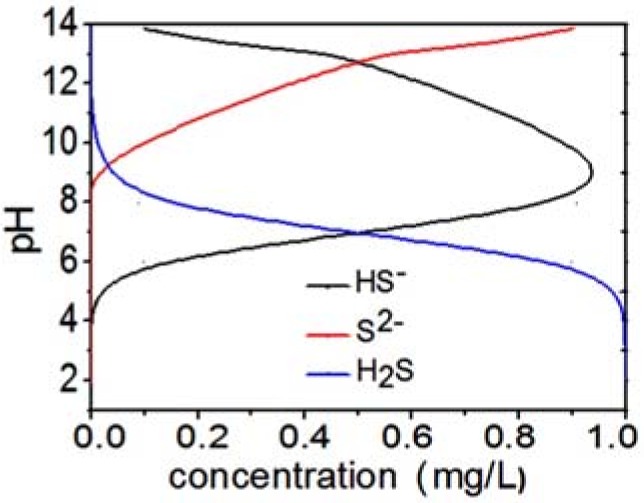
The equilibrium distribution of S^2−^, HS^−^ and H_2_S in various pH media.

Sulfide solution contains three substances: S^2−^, HS^−^ and H_2_S, existing together (Figure 1). S^2−^, as a weak acidic anion, is easy hydrolyzed to HS^−^ and H_2_S in aqueous solution. The concentrations of S^2−^, HS^−^ and H_2_S in the sulfide solutions depend on the degree of hydrolysis of sulfide which has a direct relationship with the pH values of the solutions [24,25,26]. The following relationship could be found by calculation: H_2_S dominates in the solution when the pH is ≤6. The main substance of the solution was HS^−^ at pH values between 7 and 13. When the pH is more than 14, almost all of the sulfur was converted into S^2−^. The optimum pH of the luminescent bacterium Q67 is from 7 to 9. The aqueous solution was turbid when the pH is ≤4 or ≥11, and the luminescence of these solutions was the same as that of double distilled water without bacteria. We inferred that the luminescent bacteria in this solution were already killed by the high concentration of H^+^ or OH^−^, so the EC_50_ values couldn’t be measured at pH 3, 4 and 11. This study was therefore focused on the toxicity of the sulfide solution in the pH range 5–10, in which S^2−^ was almost non-existant. As shown in Figure 1, the main sulfide was H_2_S at pH 5 to 6.5, and HS^−^ at pH 7 to 11. 

The experimental EC_50_ values at various pH media are given in Table 1. 

**Figure 2:**
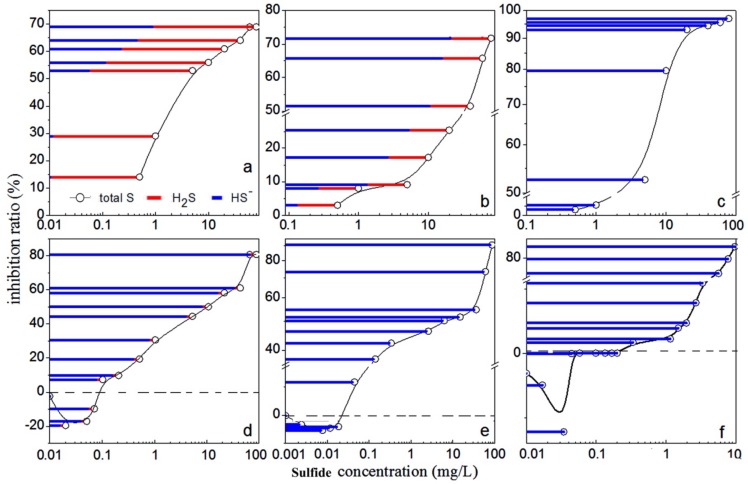
The relationship between the inhibition ratio and sulfide concentration in various pH media: (**a**) pH 6; (**b**) pH 6.5; (**c**) pH 10; (**d**) pH 7.4; (**e**) pH 8; and (**f**) pH 9.

The effect of the diverse sulfide forms in the sulfide solution on luminous bacteria Q67 at different pH values were plotted in Figure 2. It was shown that the relationship between the light emission and the sulfide concentration displayed an inverse exponential dose-response at pH 5, 6.5 and 10 (Figure 2a–c). The low-dose stimulation and a high-dose inhibition appeared at pH 7.4, 8 and 9 (Figure 2d–f), in which [HS^−^] >> [H_2_S] > [S^2−^]. The error range was less than 5%.

As shown in Table 1, the EC_50_ values of sulfide were 1.68 mg/L at pH 5 and 8.32 mg/L at pH 10, which were less than those at pH 7 to 9. The toxicity of this solution almost coincided with the concentration of H_2_S at pH 5, *i.e.*, the toxicity depended on H_2_S. Similarly, the toxicity of sulfide depended on HS^−^ at pH 10 (Figure 2c). Since the EC_50_ value at pH 5 was lower than that at pH 10, the toxicity of H_2_S could be higher than HS^−^.

However, it was difficult to compare the EC_50_ values of sulfide obtained in this study to the values reported in the literature due to the limited information about the toxicity of soluble sulfide on luminous bacteria found. The EC_50_ values were close (Table 1) and the toxicity of sulfide solution and HS^-^ almost coincided at pH 7.4, 8.0 and 9.0 (Figure 2d–f). This indicated that pH itself had little effect on the light emission of luminescent bacteria within the optimum pH range. An explanation for this was that, as many microorganisms do, luminescent bacterium was able to maintain its internal pH when the external pH changed [27,28]. 

From Figure 2a–c inverse exponential dose-response relationships were observed at pH 5, 6.5, and 10. The light emission decreased rapidly with the increasing sulfide concentration, and then tended to be constant. A possible reason was that toxicant destroyed irreversibly the bioluminescence system enzyme [29]. The influence of xenobiotics on enzyme bioluminescence systems could be described in terms of effects on the primary physicochemical processes: electron and proton (e^−^, H^+^) transfer, and the physicochemical characteristics of the compounds specify the changes in bioluminescence emission kinetics [30]. The bioluminescence emitted by luminescent bacteria comes from the nicotinamide adenine dinucleotide phosphate [NAD(P)H]-mediated respiratory electron-transport chain [31]. H_2_S could produce a reversible inhibition of the NADH oxidase activity [32] which might inhibit respiratory electron flow from NAD(P)H to flavin mononucleotide (FMN), and preventing the turnover of oxyluciferin (FMN) to luciferin (FMNH_2_). This would result in decreased light emission from the luminescent bacteria [33].

It must be pointed out that at pH 7.4, 8 and 9 (Figure 2d–f), the sulfide solution caused a typical biphasic dose-response phenomenon called hormesis-effect that was characterized by a low-dose stimulation and a high-dose inhibition [34,35,36,37]. However, the mechanisms underlying hormesis induced by environmental agents are not well elucidated [38]. The current explanation about hormesis was that it is due to an overcompensation in response to a disruption in homeostasis and this was supported through experimentation [39]. The hormetic responses has an initial inhibitory response has has usually been reported, followed by a compensatory response that would happened when the organism was stimulated by external environmental pollutants. The compensatory response may eventually exceed the performance of the controls, resulting in the net stimulatory response commonly referred to as a hormetic effect [40]. This response to a temporal disruption in homeostasis may had an advantage to adapt to low levels of biological stress which was sufficient to elicit an overcompensation, however, higher levels were less likely to affect the organism after this initial exposure [41]. Under these stressed conditions the organisms must repair the stress-induced damage to ensure survival, and so overcompensating activities would ensure that enough repair was completed to accomplish this, until homeostasis was reached once again [38]. Recently no mechanisms underlying hormesis induced by sulfide solution have been reported, but one paper illustrated that sulfide was a substrate for the mitochondrial electron transport chain in mammals at even lower concentrations [42], so we speculate that sulfide as a substrate could stimulate the vital movement of bacteria at low concentration, and then promote the light emission. Although the mechanisms of action of soluble sulfide solutions on luminescent bacteria cannot be revealed from these experiments, H_2_S as a gasotransmitter rapidly travels through cell membranes without utilizing specific transporters and exerts a host of biological effects on a variety of biological targets resulting in a variety of biological responses [43,44,45,46,47]. HS^−^ goes through cell membranes less easily than H_2_S does. To permeate the membrane, HS^−^ had to pick up a proton at the membrane surface and release a proton intracellularly [48]. Even if it existed, transport of HS^−^ through Cl^−^ channels or other anion channels is not likely to play a physiologically relevant role.

## 3. Experimental

### 3.1. Materials

Na_2_S·9H_2_O (98%) was purchased from Shanghai TongYa Chemical Industry Science and Technology Co. Ltd. NaOH and HCl (Sinopharm Chemical Reagents Co. Ltd.) was used to adjust pH values. The water used was double distilled water, passed through a reverse osmosis system and further treated with a Hitech-K flow water purification system. 

### 3.2. Toxicity Tests

For the acute toxicity test, luminescent bacterium Q67 freeze-dried particles (Patent No. ZL 97 1 06203.X) was purchased from Beijing Hammatsu Photon Techniques Inc. The optimum pH range of luminescent bacterium Q67 was 7 to 9. First, bacteria freeze-dried as pellets in glass bottles were removed from −20 °C storage. Then recovery liquid (0.8% NaCl) was added and bacteria were rehydrated at 20 °C for 15 min. The standard methods for culture medium preparation and Q67 incubation are referenced to previous articles [49,50,51].

Toxicity of soluble sulfide was evaluated by measuring the inhibition of bioluminescence of luminescent bacterial strains. Dilutions of Na_2_S (0.01 to 80 mg/L) at pH 3, 4, 5, 6.5, 7.4, 8, 9, 10 and 11 that adjusted by NaOH (0.1 mol/L), HCl (0.1 mol/L) were prepared before the toxicity detection. The concentration of different forms of sulphur could be found by calculation:

$$H_{2}S↔H^{+}+{\mathrm{HS}}^{-},K_{a1}=\frac{\left[H^{+}]\cdot [{\mathrm{HS}}^{-}\right]}{\left[H_{2}S\right]}=1.1\times 10^{-7}$$

$${\mathrm{HS}}^{-}↔H^{+}+S^{2-},K_{a2}=\frac{\left[H^{+}]\cdot [S^{2-}\right]}{\left[{\mathrm{HS}}^{-}\right]}=1.26\times 10^{-13}$$

where [H^+^], [HS^−^] and [H_2_S] means the concentrations of H^+^, HS^−^ and H_2_S [28,52,53]. The luminescence inhibition assay was performed in test tubes using a luminometer (Model RS9901, Shanghai Rongsheng Biological Electronics Co.). For each test, 10 test tubes were prepared, eight for different concentration samples, one for blank control (recovery liquid) and one for pH control (only the pH was adjusted to a certain value without sulfide solution in it). Sample or control liquid (2 mL) was added into each tube, and the bacterial suspension (50 μL) was added at 10 s intervals. After 15 min exposure [19,20,21] of the bacteria to the sample at 20 °C, the relative light unit (RLU) of luminescent bacterium Q67 was measured, and the acute toxicity of the sample on Q67 was expressed as an inhibition ratio, calculated by the following expression [28]:

$$X(\%)=(1-\frac{\mathrm{LU}}{{\mathrm{LU}}_{0}})\times 100\%$$

where *LU_0_* was the RLU of Q67 exposed to the pH control and *LU* was the RLU of the same concentration samples. EC_50_ values were calculated to express the toxicity of sulfide. EC_50_ is the concentration of toxicant that produces 50% inhibition of light emission from a specific strain of bioluminescent bacteria. Each test was repeated three times, and the average inhibition ratio was taken as final result. To avoid as far as possible sulfide solution contact with oxygen, a Parafilm membrane should be added to seal the test tubes during the sulfide reaction stage. Moreover, turbulent mixing should be avoided during the tests, for example, using pipette for aspiration instead of shaking when the reaction solution need be mixed.

## 4. Conclusions

Soluble sulfide solution at various pH values showed different toxicity on luminescent bacteria Q67. This work demonstrated that light emission rapidly decreased as sulfide concentration increased in strongly acidic solution where H_2_S dominated or alkaline solution where HS^−^ dominated, then tended to be constant, which displayed an inverse exponential dose-response relationship. A hormesis-effect occurred in alkaline solutions in which the concentration of the sulfides in the solution was HS^−^ >> H_2_S > S^2−^. The most toxic substance was H_2_S in the soluble sulfide solution. The low-dose soluble sulfide would stimulate the light emission of luminescent bacteria in alkaline solution where HS^−^ dominated. From the experiment, H_2_S might be the substance that induced the toxicity to luminescent bacteria. On the contrary, it is possible for HS^-^ to induce the stimulation. The results provided a useful approach for further demonstrating mechanism of soluble sulfide interacting with organisms.

## Figures and Tables

**Table 1:** EC_50_ values of sulfide to Q67 with the concentration distribution in various pH media.

pH	Total S, mg/L	H_2_S, mg/L	HS^−^, mg/L
5.0	1.68	1.66	0.002
6.5	45.30	33.62	11.68
7.4	33.19	8.78	22.4
8.0	34.20	2.85	31.35
9.0	38.89	0.35	38.35
10.0	8.32	0.01	8.31

## References

[1] Al T.A., Martin C.J., Blowes D.W. (2000). Carbonate-mineral/water interactions in sulfide-rich mine tailings. Geochim. Cosmochim. Acta.

[2] Komori K., Miyajima S., Tsuru T., Fuji T., Mohri S., Ono Y., Sakai Y. (2009). A rapid and simple evaluation system for gas toxicity using luminous bacteria entrapped by a polyion complex membrane. Chemosphere.

[3] Bouillaud F., Blachier F. (2011). Mitochondria and sulfide: A very old story of poisoning, feeding, and signaling?. Antioxid. Redox. Signal..

[4] Earson J., Havill D.C. (1988). The effect of hypoxia and sulphide on culture-grown wetland and non-wetland plants. II. Metabolic and physiological changes. Exp. Bot..

[5] Pezhki S.R., Pan S.Z., Delaune R.D. (1988). Sulfide-induced toxicity Inhibition of carbon assimilation in Spartina alterniflora. Photosynth. Res..

[6] Och M.S., Mendelssohn L.A. (1989). Sulphide as a soil phytotoxin: Differential responses in two marsh species. J. Ecol..

[7] Och M.S., Mendelssohn L.A., Mckee K.L. (1990). Mechanism for the hydrogen sulfide-induced growth limitation in wetland macrophytes. Limnol. Oceanogr..

[8] Prader A., Raymond A.P. (1983). Adenine ncleotide ratios and adenylate energy charge in energy metabolism. Ann. Rev. Plant. Physiol..

[9] Cao Y., Wang H.-J., Cao C., Sun Y.-Y., Yang L., Wang B.-Q., Zhou J.-G. (2011). Inhibition effects of protein-conjugated amorphous zinc sulfide nanoparticles on tumor cells growth. J. Nanopart. Res..

[10] Prader A., Raymond A.P. (1983). Adenine nncleotide ratios and adenylate energy charge in energy metabolism. Ann. Rev. Plant Physiol..

[11] Sleber M., Braendle R. (1991). Energy metabolism in rhizomes of *Acorus calamus* (L.) and in tubers of *Solanum tuberosum* (L.) with regard to their anoxia tolerance. Bot. Acta.

[12] Eberhard K.S., Falk D., Rolf A. (2005). Effects of hydrogen sulfide to vibrio fischeri, scenedes musvacuolatus, and daphnia magna. Environ. Toxicol. Chem..

[13] Julian D., Dalia W.E., Arp A.J. (1998). Neuromuscular sensitivity to hydrogen sulfide in the marine invertebrate Urechis caupo. J. Exp. Biol..

[14] Arbuckle W.B., Alleman J.E. (1992). Effluent toxicity testing using nitrifiers and Microtox^TM^. Water Environ. Res..

[15] Guidotti T.L. (1999). Hydrogen sulphide. Occup. Med. (Lond).

[16] Toussaint M.W., Shedd T.R., Vander Schaile W.H., Leather G.R. (1995). A comparison of standard acute toxicity tests with rapid-screening toxicity tests. Environ. Toxicol. Chem..

[17] Ma M., Tong Z., Wang Z., Zhu W. (1999). Acute toxicity bioassay using the freshwater luminescent bacterium *Vibrio qinghaiensis* sp. Nov.-Q67. Bull. Environ. Contam. Toxicol..

[18] Shen K.L., Shen C.F., Lu Y., Tang X.J., Zhang C.K., Chen X.C., Shi J.Y., Lin Q., Chen Y.X. (2009). Hormesis response of marine andfreshwater luminescent bacteria to metal exposure. Biol. Res..

[19] Zhang J., Liu S.S., Liu H.L. (2009). Effect of ionic liquid on the toxicity of pesticide to *Vibrio-qinghaiensis* sp.-Q67. J. Hazar. Mater..

[20] Zhao H.M., Zhang C.Y., Ge Z.G., Wang Z.Y. (2010). Toxicity measurement of the fluorobenzene derivants against *Vibrio Qinghaiensis* (Q67) and their 2D, 3D-QSAR study. Chin. J. Struct. Chem..

[21] Zhou X.F., Sang W.J., Liu S.S., Zhang Y.L., Ge H.L. (2010). Modeling and prediction for the acute toxicity of pesticide mixtures to the freshwater luminescent bacterium *Vibrio qinghaiensis* sp.-Q67. J. Environ. Sci..

[22] Salizzato M., Bertato V., Pavoni B., Volpi Ghirardini A., Ghetti P.F. (1998). Sensitivity limits and EC_50_ values of the *Vibrio fischeri* test for organic micropollutants in natural and spiked extracts from sediments. Environ. Toxicol. Chem..

[23] Johnson T., Laise C., Férard J.F. (2005). Small-scale Freshwater Toxicity Investigations—Microtox® Acute Toxicity Test.

[24] Hargrave B.T., Holmer M., Newcombe C.P. (2008). Mechanism for the hydrogen sulfide-induced growth limitation in wetland macrophytes. Mar. Pollut. Bull..

[25] Lavilla I., Pena-Pereira F., Gil S., Costas M., Bendicho C. (2009). Microvolume turbidimetry for rapid and sensitive determination of the acid labile sulfide fraction in waters after headspace single-drop microextraction with in situ generation of volatile hydrogen sulfide. Anal. Chim. Acta.

[26] van den Bosch P.L.F., van Beusekom O.C., Buisman C.J.N., Janssen A.J.H. (2007). Sulfide oxidation at halo-alkaline conditions in a fed-batch bioreactor. Biotechnol. Bioeng..

[27] Padan E., Schuldiner S. (1986). Intracellular pH regulation in bacterial cells. Meth. Enzymol..

[28] Krulwich T.H., Ito M., Gilmore R., Guffanti A.A. (1997). Mechanisms of cytoplasmic pH regulation in alkaliphilic strains of Bacillus. Extremophiles.

[29] Johnson F.H., Eyring H., Steblay R., Chaplin H., Huber C., Gherardi G. (1945). The nature and control of reactions in bioluminescence: With special reference to the mechanism of reversible and irreversible inhibitions by hydrogen and hydroxyl ions, temperature, pressure, pressure, alcohol, urethane, and sulfanilamide in bacteria. J. Gen. Physiol..

[30] Kudryasheva N.S. (1999). Mechanisms of the effect of xenobiotics on bacterial bioluminescence. Luminescence.

[31] Bose J.L., Kim U., Bartkowski W., Gunsalus R.P., Overley A.M., Lyell N.L., Visick K.L., Stabb E.V. (2007). Bioluminescence in *Vibrio fischeri* is controlled by the redox-responsive regulator ArcA. Mol. Microbiol..

[32] Samhan-Arias A.K., Garcia-Bereguiain M.A., Gutierrez-Merino C. (2009). Hydrogen sulfide is a reversible inhibitor of the NADH oxidase activity of synaptic plasma membranes. Biochem. Biophys. Res. Commun..

[33] Wang W., Nykamp J., Huang X.D., Gerhardt K., Dixon D.G., Greenberg B.M. (2009). Examination of the mechanism of phenanthrenequinone toxicity to *Vibrio fischeri*: Evidence for a reactive oxygen species-mediated toxicity mechanism. Environ. Toxicol. Chem..

[34] Yakovlev A.Y., Tsodikov A.D., Bass L.A. (1993). Stochastic model of hormesis. Math. Biosci..

[35] Bounias M., Navonenectoux M., Popeskovic D.S. (1995). Toxicology of cupric salts in honeybees. I. Hormesis effects of organic derivatives on lethality parameters. Ecotoxicol. Environ. Saf..

[36] Bounias M., Kruk I., Nectoux M., Popeskovic D. (1996). Toxicology of cupric salts on honeybees. 5. Gluconate and sulfate action on gut alkaline and acid phosphatases. Ecotoxicol. Environ. Saf..

[37] Jiang G.F., Duan W.X., Xu L., Song S.Z., Zhu C.C., Wu L. (2009). Biphasic effect of cadmium on cell proliferation in human embryo lung fibroblast cells and its molecular mechanism. Toxicol. In Vitro.

[38] Ren H.W., Shen J.W., Tomiyama-Miyaji C., Watanabe M., Kainuma E., Inoue M., Kuwano Y., Abo T. (2006). Augmentation of innate immunity by low-dose irradiation. Cell. Immunol..

[39] Calabrese E., Howe K. (1976). Stimulation of growth of peppermint (*Mentha piperita*) by Phosfon, a growth retardant. Physiol. Plant..

[40] Calabrese E.J. (1999). Evidence that hormesis represents an ‘‘overcompensation’’ response to a disruption in homeostasis. Ecotoxicol. Environ. Saf..

[41] Stebbing A.R.D. (1997). A theory for growth hormesis. Belle Newsl..

[42] Earson J., Havill D.C. (1988). The effect of hypoxia and sulphide on culture-grown wetland and non-wetland plants II. Metabolic and physiological changes. Exp. Bot..

[43] Calabrese E., Baldwin L. (1999). Reevaluation of the fundamental dose response relationship. Bioscience.

[44] Fiorucci S., Distrutti E., Cirino G., Wallace J.L. (2006). The emerging roles of hydrogen sulfide in the gastrointestinal tract and liver. Antioxid. Redox Signal..

[45] Szabo C. (2007). Hydrogen sulphide and its therapeutic potential. Nat. Rev. Drug Discov..

[46] Calvert J.W., Coetzee W.A., Lefer D.J. (2010). Novel insights into hydrogen sulfide-mediated cytoprotection. Antioxid. Redox Signal..

[47] Figura M., Chilton L., Liacini A., Viskovic M.M., Phan V., Knight D. (2009). Blockade of K(ATP) channels reduces endothelial hyperpolarization and leukocyte recruitment upon reperfusion after hypoxia. Am. J. Transplant..

[48] Mathai J.C., Missner A., Kügler P., Saparovb S.M., Zeidel M.L., Lee J.K., Pohl P. (2009). No facilitator required for membrane transport of hydrogen sulfide. Proc. Natl. Acad. Sci. USA.

[49] Liu B.Q., Ge H.L., Liu S.S. (2006). Microplate luminometry for toxicity bioassay of environmental pollutant on a new type of fresh water luminescent bacterium (*Vibrio qinghaiensis* sp.-Q67). Asian J. Ecotoxicol..

[50] Ma M., Tong Z., Wang Z., Zhu W. (1999). Acute toxicity bioassay using the freshwater luminescent bacterium *Vibrio qinghaiensis* sp. Nov.-Q67. Bull. Environ. Contam. Toxicol..

[51] Zhang Y.H., Liu S.S., Song X.Q., Ge H.L. (2008). Prediction for the mixture toxicity of six organophosphorus pesticides to the luminescent bacterium Q67. Ecotoxicol. Environ. Saf..

[52] Hargrave B.T., Holmer M., Newcombe C.P. (2008). Mechanism for the hydrogen sulfide-induced growth limitation in wetland macrophytes. Mar. Pollut. Bull..

[53] Lavilla I., Pena-Pereira F., Gil S., Costas M., Bendicho C. (2009). Microvolume turbidimetry for rapid and sensitive determination of the acid labile sulfide fraction in waters after headspace single-drop microextraction with in situ generation of volatile hydrogen sulfide. Anal. Chim. Acta.

